# Posttraumatic midshaft clavicular shortening does not result in relevant functional outcome changes: Observations in 32 healed, nonoperatively treated fractures

**DOI:** 10.3109/17453674.2015.1040982

**Published:** 2015-09-04

**Authors:** Sylvia A Stegeman, Pieter Bas de Witte, Sjoerd Boonstra, Jurriaan H de Groot, Jochem Nagels, Pieta Krijnen, Inger B Schipper

**Affiliations:** ^1^Department of Trauma Surgery; ^2^Department of Orthopaedics; ^3^Department of Rehabilitation Medicine; ^4^the Laboratory for Kinematics and Neuromechanics, Leiden University Medical Center, Leiden, the Netherlands.

## Abstract

**Background and purpose:**

Shoulder function may be changed after healing of a nonoperatively treated clavicular fracture, especially in cases of clavicular shortening or mal-union. We investigated scapular orientations and functional outcome in healed clavicular fractures with and without clavicular shortening.

**Patients and methods:**

32 participants with a healed nonoperatively treated midshaft clavicular fracture were investigated. Motions of the thorax, arm, and shoulder were recorded by standardized electromagnetic 3D motion tracking. The DASH score and Constant-Murley score were used to evaluate functional outcome. Orientation of the scapula and humerus at rest and during standardized tasks, and strength and function of the affected shoulders were compared with corresponding values for the uninjured contralateral shoulders.

**Results:**

Mean clavicular shortening was 25 mm (SD 16). Scapula protraction had increased by mean 4.4° in rest position in the affected shoulders. During abduction, slightly more protraction, slightly more lateral rotation, and slightly less backward tilt was found for the affected shoulders. For anteflexion, the scapular orientations of the affected shoulders also showed slightly increased protraction, slightly increased lateral rotation, and slightly reduced backward tilt. Scapulohumeral kinematics, maximum humerus angles, and strength were not associated with the degree of clavicular shortening. All participants had excellent performance on the Constant-Murley score and DASH score.

**Interpretation:**

Scapulohumeral kinematics in shoulders with a healed clavicular fracture differ from those in uninjured shoulders, but these changes are small, do not result in clinically relevant changes in outcome, and do not relate to the amount of clavicular shortening. These findings do not support routine operative reduction and fixation of shortened midshaft clavicular fractures based on the argument of functional outcome.

Displaced midshaft clavicular fractures are often treated nonoperatively with good results, despite the frequent presence of initial clavicular shortening (Eskola et al. 1986, Nordqvist and Petersson 1994, Robinson 1998, Hillen et al. 2010). Studies on clinical outcome after clavicular shortening have given conflicting results: some have shown shortening to be associated with poor functional outcome (Eskola et al. 1986, Hill et al. 1997, Lazarides and Zafiropoulos 2006), whereas others have indicated no such relationship (Nordqvist et al. 1997, Oroko et al. 1999, Nowak et al. 2005, Rasmussen et al. 2011). Mal-union of the clavicle leads to an altered position of the scapula relative to the thorax (Ledger et al. 2005, Veeger and van der Helm 2007), which may cause shoulder problems such as acromioclavicular osteoarthritis, reduced arm-shoulder functionality, and symptomatic winging of the scapula (Ledger et al. 2005, Hillen et al. 2012, Ristevski et al. 2013). Primary operative treatment may therefore be preferred in patients with substantial clavicular shortening (Canadian Orthopaedic Trauma Society 2007), or to prevent non-union (McKee et al. 2012). Operative treatment of clavicular midshaft fractures has become more common (Stegeman et al. 2013). However, the influence of shortening on clavicular and scapulohumeral movement and on functional outcome has not been sufficiently studied to substantiate the need for primary operative reduction and fixation of displaced clavicular fractures in order to prevent poor functional outcome.

Our main goal was to assess scapular orientation and arm-shoulder kinematics in patients with healed nonoperatively treated midshaft clavicular fracture, and to compare this to their uninjured contralateral shoulder. A secondary goal was to assess the relationship between clavicular shortening and scapular orientation, and between clavicular shortening and functional outcome.

## Patients and methods

### Inclusion criteria and participants

No sample size calculation was performed. 30 participants were considered sufficient for this exploratory study. Eligible candidates who had sustained a unilateral, nonoperatively managed midshaft clavicular fracture that had healed within 4 months were selected from the medical databases from 2006–2010 at the Leiden University Medical Center and the Rijnland Hospital in the Netherlands. Further inclusion criteria were age between 18 and 60 years and no associated injuries at the time of trauma. Exclusion criteria were pathological fractures, neurovascular injury and other conditions influencing arm and shoulder function of either the affected arm or the contralateral arm, current or previous acromioclavicular (AC) injury such as AC luxation or symptomatic AC osteoarthritis not caused by the clavicular fracture, and a fracture in the proximal or distal third of the clavicle. Since an electromagnetic field was used in this study, candidates with a cardiovascular pacemaker were also excluded. All 74 eligible candidates received written information on the study and were subsequently contacted by telephone, and 32 were willing to participate.

### Motion recording

**Figure 1.**
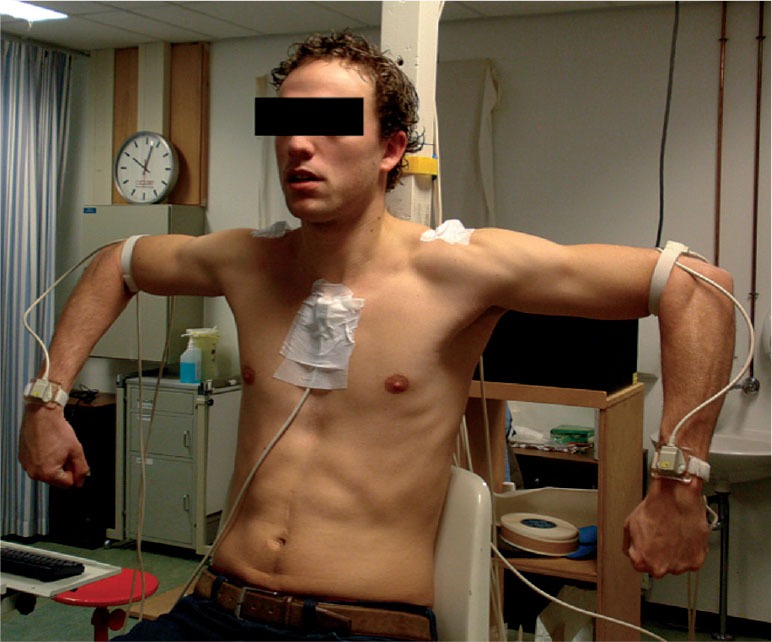
Positioning of the sensors during maximum internal rotation.

To collect 3D motion data of the arm and scapula with respect to the thorax, the “Flock of Birds” 3D Electromagnetic Motion Tracking Device (FoB; Ascension Technology Corp., Burlington, VT) and specialized computer software for skeletal motion (FOBVis; Clinical Graphics, Delft, the Netherlands) were used. The FoB motion sensors were taped to the skin covering the posterolateral surface of the acromion, the sternum, both arms on the posterior aspect just proximal to the humeral epicondyles, and the wrist (Figure 1). Another sensor was used to localize standardized predefined bony landmarks in 3D relative to the other sensors. The sensors were positioned by the main researcher in a standardized way. The center of the glenohumeral joint was determined using a regression method. The landmarks recorded were used to create 3D local bone coordinate systems, based on individual anatomy of the participants (Meskers et al. 1998). For this purpose, the International Society of Biomechanics (ISB) definitions of joint coordinate systems were used (Wu et al. 2005). Samples were taken at a sample rate of ± 30 Hz.

**Figure 2.**
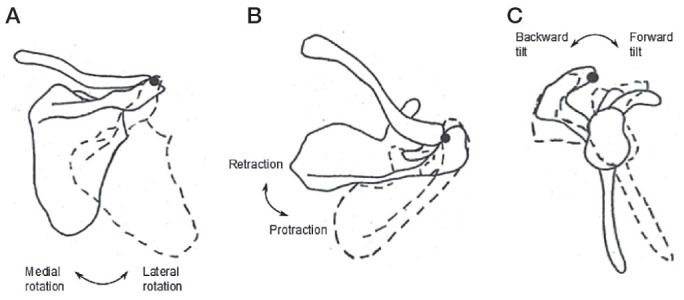
Scapular orientation. We adapted the terminology used in the original figure in (A) from downward rotation / upward rotation to medial rotation/ lateral rotation, in (B) from external rotation / internal rotation to retraction / protraction, and in (C) from posterior tilting/anterior tilting to backward tilt/forward tilt. Figures reprinted with permission from Borich et al. J Orthop Sports PhysTher 2006; 36: 926-934. http://dx.doi.org/10.2519/jospt.2006.2241.

Participants were asked to perform a number of standardized tasks with both arms while seated with their trunk in erect position and the hip and knees flexed about 90°. Firstly, scapular orientation was measured at rest, expressed in degrees of protraction, lateral rotation, and backward tilt (Figure 2). By convention, protraction means anterior rotation of the lateral border of the scapula; lateral rotation means lateral rotation of the inferior angle; backward tilt means that the scapula rotates in such a way that the cranial border of the scapula moves dorsally (Wu et al. 2005). Secondly, maximum angles of humerus exertions relative to the thorax were measured for abduction (AB), anteflexion (AF), retroflexion (RF), and internal and external rotation of the humerus with the arm at 90° of abduction and with 0° of horizontal abduction (Figure 1). Thirdly, scapular orientations (protraction, lateral rotation, and backward tilt) during AB and AF were measured. All measurements were acquired for both arms simultaneously, whereas the contralateral unaffected shoulder acted as the control shoulder.

### Clinical outcome

The strength of both arms was tested with a hand-held dynamometer (MicroFET2; Hoggan Health Industries Inc., West Jordan, UT). To measure maximum force (N), the Make test was used, in which the examiner holds the dynamometer stationary while the participant exerts a maximum force against the dynamometer and examiner (Stratford and Balsor 1994). The dynamometer was placed at the medial side of the elbow joint to measure strength during adduction, 1–2 cm above the elbow joint at the lateral side for AB, anterior to the elbow (distal of the upper arm) for AF, posterior to the elbow for RF, and on the ventral and dorsal side of the wrist for subsequent external and internal rotation, while the participant was seated with the elbow flexed at 90°.

Objective functional outcome was measured using the Constant-Murley score, which ranges from 0 (worst function) to 100 (best function). The scores for the affected shoulders were adjusted for gender and age (in decades) to obtain relative Constant scores, which were compared with published reference values for the general population. Subjective functional outcome was measured using the Disabilities of the Arm, Shoulder and Hand (DASH) score. A lower DASH score indicates less disability and dysfunction. The scores were compared to reference values (Hunsaker et al. 2002).

### Radiography

Clavicular shortening was expressed as a proportion of the total clavicular length before fracture, in order to obtain a relative measure that accounts for inter-individual variation in clavicular length. The length before fracture was calculated by adding the length of the affected clavicle to the amount of measured fracture overlap, as we did not have information of the length of the clavicle prior to fracture. The contralateral clavicle was not used as a reference, because of possible pre-existent clavicular asymmetry (Cunningham et al. 2013, Wisanuyotin et al. 2013). To calculate this relative shortening, the initial anteroposterior (AP) trauma radiograph was used—as well as an AP panorama radiograph comprising both clavicles that was acquired during the study visit (i.e. after consolidation) of all participants. It was ensured that the participants were standing straight and that the spinous processes of the thoracic vertebrae were projected in the midline, to eliminate thoracic rotation and clavicular protraction on the panorama radiograph. On both radiographs, the length of the affected clavicle was digitally measured as the straight line between the mid-medial border of the sternoclavicular (SC) joint and the most lateral edge of the acromioclavicular (AC) joint. Overlap of fracture fragments was measured on the trauma radiograph as the axial distance between the ends of the cortical fragments. As a measure of relative shortening, the clavicle shortening index after fracture consolidation (CSI_cons_) was calculated as follows:

Eq. 1
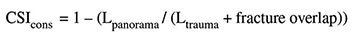


In which L_trauma_ is the length of the affected clavicle after trauma, fracture overlap is the overlap between the fracture fragments measured on the trauma radiograph, and L_panorama _is the length of the consolidated affected clavicle. This equation is an adjustment of the equation proposed by Smekal et al. (2008).

### Statistics

Scapular orientation at rest and maximum humerus angles of the affected shoulders were compared to those of the control shoulders using paired t-tests. The relationship between clavicular shortening (CSI_cons_) and scapular orientation and maximum humerus angles was assessed using linear regression analysis. If a statistically significant association between CSI_cons_ and scapular orientation and maximum humerus angles was found, an interaction term with arm dominance was tested.

Scapular orientations during AB and AF were plotted for the complete range of motion. In the analysis of scapular orientation during AB and AF, measurements above 90°of humerus elevation were not included, because above 90° the accuracy of FoB acromion sensor recording is known to be reduced due to skin and soft tissue motion artifacts (Meskers et al. 2007). The association between humerus elevation and scapular orientation was analyzed using linear mixed models with a random effect per subject to account for repeated measures. To determine whether the association between humerus elevation angle and scapular orientation was non-linear, a squared term for humerus elevation angle was tested and was included in the model if statistically significant. To analyze whether scapular orientation during AB and AF differed between the affected and contralateral shoulders, side (control vs. affected) was also included as independent variable in the mixed models. To test whether the difference in scapular orientation between the affected and contralateral shoulders was constant during AB and AF, an interaction term between side and humerus elevation angle was tested in each model and included if statistically significant. To illustrate the effect of humerus elevation angle on scapular orientation during AB and AF, the model’s predicted values for scapular orientation are plotted for the affected and control shoulders. Also, predicted values for scapular orientation at 15°, 30°, 60°, and 90° of humerus elevation for the affected and contralateral shoulders are tabulated for illustrative purposes. To assess the association between clavicular shortening and scapular orientation of the affected shoulder during AB and AF, similar linear mixed models were fitted for only the affected shoulders, with CSI_cons_ as independent variable.

Arm strength was compared between the affected and contralateral arms using paired t-tests. Linear regression analyses were performed to estimate the influence of CSI_cons_ on AB and AF strength.

All statistical analyses were performed using SPSS version 20.0. Any p-values < 0.05 were considered statistically significant.

### Ethics and registration

Approval for this exploratory study was obtained from the medical ethics review committee of Leiden University Medical Center, the Netherlands. Each participant provided written informed consent. The study was registered in the Dutch Trial Registry (NTR3167) as an observational study and was conducted between December 2011 and April 2012. The study is reported according to the STROBE statement for observational studies (von Elm et al. 2007).

## Results

32 subjects with a history of a midshaft clavicular fracture participated in the study (median age 31 (21–62) years, 27 males) (Table 1). 30 of the participants were right-handed, and in 15, the consolidated clavicular fracture was on the dominant side. Mean clavicular shortening after consolidation was 25 (SD 16) mm and mean CSI_cons_ was 0.13 (SD 0.08). For 1 patient, the CSI_cons_ could not be calculated because the trauma radiograph had not been calibrated.

**Table 1.** Demographic characteristics of the 32 participants

Parameter	Total	Male	Female
	n = 32	n = 27	n = 5
Age in years, median (range)	31 (21–62)	36 (21–62)	27 (25–31)
Side of fracture, n			
Right	16	13	3
Dominant side affected, n			
Yes	15	12	3
Shortening in mm after consolidation, mean (SD)	25 (16)	26 (16)	16 (19)
Clavicle shortening index, mean (SD)	0.13 (0.08)	0.14 (0.07)	0.09 (0.11)
Trauma mechanism, n			
Bicycle	15	12	3
Traffic (motorized vehicles)	6	5	1
Sports injury	7	6	1
Other	4	4	0
Occupation, n			
Manual worker	12	10	2
Office work	19	16	3
Unemployed	1	1	0
Current complaint, n			
None	13	11	2
Crepitation	4	4	0
Irritation/weariness	13	10	3
Pain	2	2	0

### Scapular orientation in rest position

In rest position, there was more scapula protraction in the affected shoulders (with a mean difference of 4.4°; p = 0.05) (Table 2). No statistically significant effect of CSI_cons_ on the rest position of the scapula was found (regression coefficient for protraction: 0.11; for lateral rotation: 0.07; and for backward tilt: −0.1; all p > 0.10).

**Table 2.** Differences between affected and contralateral (control) arms for scapular orientation in rest position and for maximum humerus angles

Task	Affected	Control	Affected vs. control	
	mean (SD)	mean (SD)	Mean diff. (95% CI)	p-value
Scapular orientation in rest position, degrees				
Protraction	28 (9.6)	24 (6.9)	4.4 (0.0 to 8.9)	0.05
Lateral rotation	3.4 (5.0)	1.8 (6.3)	1.6 (–0.9 to 4.1)	0.2
Backward tilt	−12 (6.4)	−11 (5.3)	−1.6 (–3.5 to 0.4)	0.1
Maximum humerus angle, degrees				
Abduction	151 (11.9)	150 (11.0)	1.0 (–1.8 to 3.8)	0.5
Anteflexion	147 (10.7)	145 (9.5)	2.1 (–0.5 to 4.6)	0.1
Retroflexion	61 (9.8)	60 (8.9)	1.0 (–1.3 to 3.3)	0.4
Internal rotation	54 (16.5)	53 (16.8)	0.9 (–3.7 to 5.5)	0.7
External rotation	70 (11.7)	72 (10.6)	–2.1 (–6.3 to 2.0)	0.3

### Maximum humerus angles

Maximum humerus angles during AB, AF, RF, internal rotation, and external rotation were similar between the affected shoulders and the control shoulders (Table 2). No statistically significant effect of CSI_cons_ on the differences in maximum humerus angle was found (regression coefficient for AB: 0.01; for AF: 0.07; for RF: −0.07; for internal rotation: −0.05; and for external rotation: −0.1; all p > 0.10).

### Scapular orientation during abduction and anteflexion

**Figure 3.**
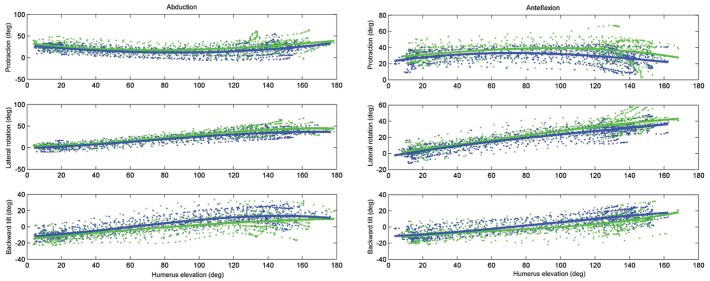
Scapular orientation during active arm abduction (left panels) in affected shoulders (green lines) and contralateral control shoulders (blue lines) and during active arm anteflexion (right panels) **^a^**. Overall, affected shoulders had more scapula protraction, more lateral rotation, and less backward tilt than the contralateral control shoulders. **^a ^**Values above 90 degrees were not included in the analysis because of possible inaccuracy.

In Figure 3, the raw values for measurements of scapular orientation during AB and AF have been plotted against humerus elevation angle.

**Figure 4.**
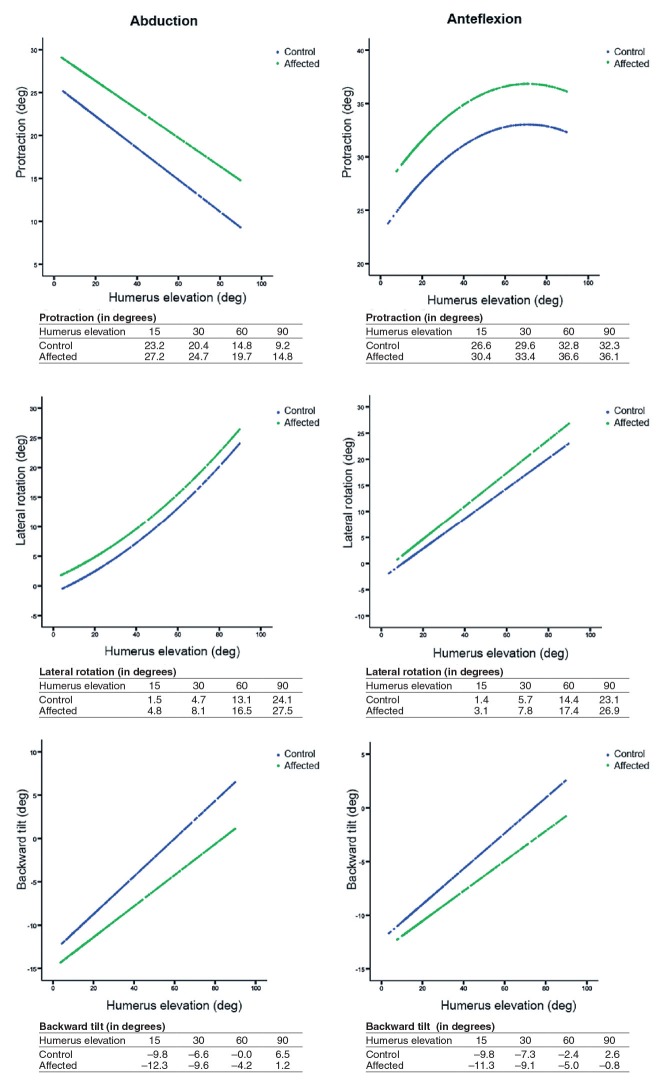
Estimated outcomes of the mixed model analyses on scapular orientation during abduction and anteflexion in affected and control shoulders.

During AB, overall scapula protraction decreased by 1.8° per 10-degree increase in humerus angle. Over the range of humerus elevation studied (0–90°), the difference in scapula protraction between the affected shoulders and the contralateral shoulders was constant (4.4°) (Figure 4 and Table 3). Lateral rotation of the scapula increased exponentially during AB for both the affected shoulders and the control shoulders. Lateral rotation of the scapula of the affected shoulder was 2.4° higher than that of the contralateral shoulder over the complete range of humerus elevation angles. Scapular backward tilt increased linearly during AB and was −1.9° lower for the affected shoulders, with a systematic increase of 2.2°. The difference between the affected shoulders and the contralateral shoulders increased by 0.4° per 10-degree increase in humerus elevation angle (Figure 4 and Table 3). No statistically significant effects of CSI_cons_ were found on the affected scapular movements for every 10° of humerus elevation for protraction (0.4°), lateral rotation (−2.4°), and backward tilt (−0.6°).

**Table 3.** Outcomes of linear mixed model analyses on scapular orientation during abduction and anteflexion

	Abduction			Anteflexion		
	Mean estimate	p-value	95% CI	Mean estimate	p-value	95% CI
Protraction						
Affected side	4.4	< 0.001	3.6 to 5.2	3.8	< 0.001	3.1 to 4.5
Humerus angle (per 10°)	−1.8	< 0.001	−1.9 to −1.6	2.9	< 0.001	2.1 to 3.7
Humerus angle squared (per 10°)	N/A	-	-	−0.02	< 0.001	−0.02 to −0.01
Affected side x humerus angle (per 10°)	N/A	-	-	N/A	-	-
Lateral rotation						
Affected side	2.4	< 0.001	2.0 to 2.8	1.3	< 0.001	0.6 to 1.9
Humerus angle (per 10°)	1.5	< 0.001	1.1 to 2.0	2.9	< 0.001	2.8 to 3.0
Humerus angle squared (per 10°)	0.01	< 0.001	0.01 to 0.02	N/A	-	-
Affected side x humerus angle (per 10°)	N/A	-	-	0.3	0.001	0.1 to 0.4
Backward tilt						
Affected side	−1.9	< 0.001	−2.6 to −1.2	−1.0	0.001	−1.7 to −0.4
Humerus angle (per 10°)	2.2	< 0.001	2.1 to 2.3	1.7	< 0.001	1.5 to 1.8
Humerus angle squared (per 10°)	N/A	-	-	N/A	-	-
Affected side x humerus angle (per 10°)	−0.4	< 0.001	−0.5 to −0.2	−0.3	0.001	−0.4 to −0.1

During AF, scapula protraction increased hyperbolically (Table 3 and Figure 4). Up to an angle of 90° of humerus elevation, protraction of the affected shoulder was constantly 3.8° higher than for the contralateral side. Lateral rotation of the scapula increased linearly during AF, and was higher for the affected shoulders. The difference in lateral rotation of the scapula between the affected shoulders and the contralateral shoulders increased by 0.3° per 10-degree increase in humerus elevation angle during AF. Scapular backward tilt increased linearly during AF. In the same way as during AB, backward tilt during AF was lower for the affected shoulders and the difference increased by 0.3° per 10-degree increase in humerus elevation angle (Table 3 and Figure 4). No statistically significant effect of CSI_cons_ on the affected scapular movements for every 10° of humerus elevation was found for protraction (−1.7°), lateral rotation (−2.6°), and backward tilt (−0.4°).

### Clinical outcome

19 of the 32 participants included reported irritation, a feeling of weariness, and pain in the affected shoulder—mostly after prolonged shoulder activity (Table 1). None of the participants were under treatment for these complaints.

No statistically significant systematic differences in arm strength between control shoulders and affected shoulders were found for adduction (mean difference 7.2 N, 95% CI: −3.5 to 18), AB (mean difference −0.10 N, 95% CI: −8.8 to 8.6), AF (mean difference 9.6 N, CI: −3.1 to 22), RF (mean difference 1.6 N, CI: −6.7 to 9.8), external rotation (mean difference 2.0 N, CI: −3.2 to 7.3), and internal rotation (mean difference 5.1 N, CI: −0.8 to 11.1). There was no association between CSI_cons_ and arm strength for all shoulder movements (adduction: beta = −1.29, p = 0.07; AB: beta = −0.47, p = 0.4; AF: beta = 0.59, p = 0.5; RF: beta = −0.08, p = 0.9; external rotation: beta = 0.08, p = 0.8; internal rotation: beta = 0.37, p = 0.3).

The mean Constant-Murley score was 96 (SD 5.3) points. All participants scored in the normal range relative to controls of the same sex and age (Constant 1986). The DASH outcome measure had an overall score of 5.2 (SD 6.3), which is low compared to the normative values of 10 (SD 14.7) (Hunsaker et al. 2002). Since all the participants scored in the normal range of values for the subjective and objective scores, additional analysis was not considered relevant.

## Discussion

In this study, we observed more scapular protraction in rest position for affected arms, elevated scapular protraction and lateral rotation, and reduced backward tilt during motion. Clavicular shortening was not related to scapular rotation or to maximum humerus angles and strength. Clinical outcomes for the affected arms were similar to those for the control arms, and were not affected by clavicular shortening.

To our knowledge, this is the first study to assess changes in scapular orientation during active motion after consolidation of clavicular fractures and in relation to clavicular shortening. A few studies have been conducted to examine the kinematics of the scapula after clavicular fracture relative to the contralateral shoulder, by means of computed tomography (CT) (Ledger et al. 2005, Ristevski et al. 2013), cadaveric dissection (Matsumura et al. 2010, Hillen et al. 2012, Matsumura et al. 2013), and computational models of shortened clavicles (Patel et al. 2012). These studies all involved static or passive anatomic measurements and smaller numbers of patients. In the present study, participants actively moved their arms symmetrically as instructed, which provided a more fluent motion of the humerus combined with scapular orientations instead of static measurements.

For scapular orientation in rest position, only an increased protraction of the scapula on the affected shoulder could be demonstrated, which was not related to clavicular shortening. This increased protraction has also been reported in other studies (Ledger et al. 2005, Hillen et al. 2012, Ristevski et al. 2013). The more profound protraction may explain some of the subjective shoulder complaints reported by some of the participants, although this could not be objectified by a subjective or objective reduction in arm strength, in range of motion, or in the outcomes of the DASH and Constant-Murley score. It is questionable whether the difference that we found between affected and control shoulders is clinically relevant. With a 95% CI of 0.0–8.9 between affected arms and control arms, this 4.4-degree difference appears to lie in the range of normal intra-individual variation (de Groot 1997). In addition, the maximum humerus angles were not influenced by the extent of clavicular shortening. These results are in accordance with those of several other studies that have tested range of motion after midshaft fractures of the clavicle (McKee et al. 2006, Canadian Orthopaedic Trauma Society 2007, Hillen et al. 2012).

In healthy subjects, 3D scapulohumeral movement during arm elevation leads to increased protraction (de Groot 1997, Meskers et al. 1998), reduced lateral rotation, and increased backward tilting of the scapula (Ludewig et al. 2009). In accordance with the findings of 2 other studies (Hillen et al. 2012, Matsumura et al. 2013), we found more protraction, more lateral rotation, and less backward tilt of the scapula in affected shoulders. We found no association between clavicular shortening and scapulohumeral movements, which contrasts with the findings of Matsumura et al. (2010), who found that during elevation of the humerus, backward tilt decreased and protraction increased significantly in the case of clavicular shortening of 10% or more. However, these data were acquired using cadavers with manually created fractures, where active motion is difficult to reproduce and pain is irrelevant. Pain could lead to coordinative dysfunction of the scapula, and in severe cases to scapula dyskinesia—which would negatively influence scapular orientation. This cannot be evaluated in cadaveric studies. In our study population, pain was not a limitation for subjective or objective functional outcome of the shoulder, although over half of the participants (when asked) complained of some irritation, pain, or a feeling of weariness in the shoulder during prolonged activity. As another explanation for the structural changes, one could speculate that changed axial rotation of the clavicle after mal-union—and not clavicular shortening—could have caused the altered 3D scapular orientations.

Changed muscular balance and altered kinematics of the closed chain mechanism of the shoulder may lead to a decrease in arm strength, especially in anteflexion, adduction, and internal rotation (Ledger et al. 2005, McKee et al. 2006). In previous studies, an association between shortening and clinical outcome was found if clavicular shortening was more than 15 mm (Eskola et al. 1986, Hill et al. 1997, Wick et al. 2001, Ledger et al. 2005, Lazarides and Zafiropoulos 2006). In contrast to these studies, we found no evidence that the affected arms had less strength than the contralateral arms, or that the amount of shortening or altered scapular orientation influenced strength. Also, both the Constant-Murley score and the DASH score were excellent for the affected arms. These results are supported by the findings of other studies (Nordqvist et al. 1997, Oroko et al. 1999, Nowak et al. 2005, Rasmussen et al. 2011). However, the lack of endurance and rapid fatigability was not tested in our participants.

Concerning the limitations of the present study, selection bias may have occurred because not all of the patients invited were willing to participate. The most frequent reason for non-participation was that the candidates were not willing to invest time to participate in research. 4 of the 74 invited candidates had moved and were lost to follow-up, 1 developed non-union, and 1 was operated in another hospital. Since the FoB required the static length of the clavicles to calculate the different angles, only former patients with a healed clavicular fracture could participate in our study. However, we do believe that this group of participants was a good representation of the total range of midshaft clavicular fracture patients at our hospitals, as all the patients who presented with a midshaft clavicular fracture at the emergency department received nonoperative treatment in that period.

For all comparisons in our study, the unaffected shoulder of the participants served as a control, because we assumed that the scapular orientations of the control shoulder had remained unchanged after the contralateral clavicular fracture. One could speculate that the position of the control shoulder might also have altered, due to the changed position of the affected side. This is known to happen in unilateral diseases, such as in stroke patients with hemiplegia (Meskers et al. 2005).

One limitation of our data analysis was that we could not obtain data on the scapular orientations achieved above 90° of anteflexion and abduction. This was due to potential errors in position of the acromion sensor caused by skin and soft tissue motion. Our conclusion is therefore only valid for arm movements up to 90°. More research is needed to assess this aspect of scapular orientation and possible functional limitations during overhead elevation (above 90°).

In conclusion, midshaft clavicular fractures tend to affect the scapulohumeral rhythm for arm movements below 90°, compared to the unaffected side, but these changes are small, do not appear to influence functional outcome of the shoulder, and do not seem to be related to the amount of clavicular shortening. It therefore seems less important than previously assumed to re-acquire the initial clavicle length for good functional outcome. On account of the clinically irrelevant changed scapulohumeral rhythm below 90° after clavicular shortening and the lack of significant differences in functional outcome compared to unaffected shoulders, we cannot support the current tendency towards more routinely operative reduction and fixation of all shortened midshaft clavicular fractures, based on these arguments. This conclusion does not include patients with an increased risk of non-union or those with a wish for early mobilization of the shoulder.
